# *Populus euphratica* XTH overexpression enhances salinity tolerance by the development of leaf succulence in transgenic tobacco plants

**DOI:** 10.1093/jxb/ert229

**Published:** 2013-10-01

**Authors:** Yansha Han, Wei Wang, Jian Sun, Mingquan Ding, Rui Zhao, Shurong Deng, Feifei Wang, Yue Hu, Yang Wang, Yanjun Lu, Liping Du, Zanmin Hu, Heike Diekmann, Xin Shen, Andrea Polle, Shaoliang Chen

**Affiliations:** ^1^College of Biological Sciences and Technology (Box 162), Beijing Forestry University, Beijing 100083, China; ^2^Institute of Genetics and Developmental Biology, Chinese Academy of Sciences, Beijing 100101, China; ^3^Büsgen-Institut, Forstbotanik und Baumphysiologie, Georg-August Universität Göttingen, Göttingen 37077, Germany

**Keywords:** Chlorophyll a fluorescence, leaf anatomy, NaCl, photosynthesis, *Populus euphratica*, root length, salt compartmentation, water-retaining capacity, xyloglucan endotransglucosylase/hydrolase gene.

## Abstract

*Populus euphratica* is a salt-tolerant tree species that develops leaf succulence after a prolonged period of salinity stress. In the present study, a putative xyloglucan endotransglucosylase/hydrolase gene (*PeXTH*) from *P. euphratica* was isolated and transferred to tobacco plants. PeXTH localized exclusively to the endoplasmic reticulum and cell wall. Plants overexpressing *PeXTH* were more salt tolerant than wild-type tobacco with respect to root and leaf growth, and survival. The increased capacity for salt tolerance was due mainly to the anatomical and physiological alterations caused by *PeXTH* overexpression. Compared with the wild type, *PeXTH*-transgenic plants contained 36% higher water content per unit area and 39% higher ratio of fresh weight to dry weight, a hallmark of leaf succulence. However, the increased water storage in the leaves in *PeXTH*-transgenic plants was not accompanied by greater leaf thickness but was due to highly packed palisade parenchyma cells and fewer intercellular air spaces between mesophyll cells. In addition to the salt dilution effect in response to NaCl, these anatomical changes increased leaf water-retaining capacity, which lowered the increase of salt concentration in the succulent tissues and mesophyll cells. Moreover, the increased number of mesophyll cells reduced the intercellular air space, which improved carbon economy and resulted in a 47–78% greater net photosynthesis under control and salt treatments (100–150mM NaCl). Taken together, the results indicate that *PeXTH* overexpression enhanced salt tolerance by the development of succulent leaves in tobacco plants without swelling.

## Introduction

Soil salinity is one of the major stress factors suppressing plant growth and development. High salt concentrations lead to water deficit and ion toxicity, which induce oxidative damage in plants (Zhu, 2001, 2003; Munns and Tester, 2008). Reducing salt uptake and accumulation is a major strategy for adapting glycophyte plants to saline environments. Being a valuable tree species that survives in saline and alkaline desert sites, *Populus euphratica* has great potential for genetic improvement in large-scale afforestation. Regenerated *P. euphratica* plants are able to cope with 300–450mM NaCl under hydroponic conditions (Gu *et al.*, 2004). Given its greater capacity to exclude salt, *P*. *euphratica* has been widely used as a model plant to elucidate the physiological and molecular mechanisms of salt tolerance in woody species (Chen *et al.*, 2001, 2002*a*, *b*, 2003; Ottow *et al.*, 2005; Junghans *et al.*, 2006; Wang *et al.*, 2007, 2008; Sun *et al.*, 2009*a*, *b*, 2010*a*, *b*; Chen and Polle, 2010; Ding *et al.*, 2010). Under salinity stress, *P*. *euphratica* diminishes NaCl loading of the xylem due to salt transport restrictions by compartmentalizing Na^+^ and Cl^–^ in root cortical vacuoles (Chen *et al.*, 2002*a*, 2003). At the cellular level, salinized *P*. *euphratica* extrudes excessive Na^+^ to the apoplast but retains K^+^ in the cytosol in order to maintain K^+^/Na^+^ homeostasis (Ottow *et al.*, 2005; Sun *et al.*, 2009*a*). To reduce salt-induced oxidative damage, *P*. *euphratica* rapidly up-regulates antioxidative enzymes to scavenge reactive oxygen species (ROS) after the onset of salt treatment (Wang *et al.*, 2007, 2008). During a prolonged period of salt stress, *P*. *euphratica* develops pronounced succulent leaves by increasing the number of cell layers and formation of large cells (Ottow *et al.*, 2005). The increased cell number and volume lead to salt dilution, which benefits the adaptation of *P*. *euphratica* to salt environments (Ottow *et al.*, 2005; Chen and Polle, 2010).

Leaf succulence is a general anatomical feature of most halophytic plants (Hameed *et al.*, 2010). For these halophytes, succulent leaves and stems exhibit an ability to store water under saline conditions (Short and Colmer, 1999; Song *et al.*, 2006). Increased succulence is considered to be a compensatory strategy for avoiding excessive salt concentrations (Albert, 1975). Sodium, rather than potassium and chloride, is important for inducing leaf succulence in halophyte plants (Wang *et al.*, 2012). However, the molecular mechanism of salt-induced succulence remains poorly understood in higher plants.

Cell and tissue morphology is regulated, in part, by cell wall loosening and rearrangement. The process is dependent, to a large extent, on wall-modifying enzymes, such as expansin, xyloglucan endotransglucosylase, and β-1, 4-glucanase (Cosgrove, 2005). Among these enzymes, xyloglucan endotransglucosylase/hydrolases (XTHs) are considered to be a vital factor controlling cell wall extensibility. The XTHs exhibit xyloglucan endotransglucosylase (XET) activity (Thompson and Fry, 2001) and/or xyloglucan endohydrolase (XEH) activity (Rose *et al.*, 2002), and are hypothesized to catalyse the splitting and/or reconnection of xyloglucan cross-links in the cellulose–xyloglucan framework of plant cell walls (Fry *et al.*, 1992; Nishitani and Tominaga, 1992). Using genome sequencing, multigene families of XTHs have been identified in different plant species, including *Arabidopsis* (Yokoyama and Nishitani, 2001), rice (Uozu *et al.*, 2000; Yokoyama *et al.*, 2004), tomato (Saladié *et al.*, 2006), wheat (Okazawa *et al.*, 1993; Y. Liu *et al.*, 2007), and poplar (Geisler-Lee *et al.*, 2006). XTH expression is tissue specific and regulated by environmental and hormonal factors (Yokoyama and Nishitani, 2001; Catalá *et al.*, 2001; Jan *et al.*, 2004; Yokoyama *et al.*, 2004, 2010; Becnel *et al.*, 2006). XTHs play essential roles in a variety of growth and differentiation processes, including primary root elongation (Osato *et al.*, 2006), hypocotyl growth (Wu *et al.*, 2005), vein differentiation (Matsui *et al.*, 2005), flower opening (Harada *et al.*, 2011), fruit ripening (Saladié *et al.*, 2006; Miedes and Lorences, 2009), petal abscission (Singh *et al.*, 2011), and wood formation (Nishikubo *et al.*, 2011). However, the function of XTH in the maintenance of plant growth under salt stress is not known.

Previous microarray results showed that salt-treated *P. euphratica* increased transcription of *PeXTH* in leaves (Supplementary Fig. S1 available at *JXB* online). The present study shows that *PeXTH* is up-regulated in leaves of salinized *P. euphratica* plants, while other members of the XTH multigene family are not induced or are less induced (Supplementary Fig. S1). Thus, *PeXTH* may contribute to salt-induced leaf succulence in *P. euphratica* (Ottow *et al.*, 2005). The *XTH* gene was cloned from *P. euphratica* leaves and transferred to a model species, *Nicotiana tabacum* cv. Wisconsin 38, to investigate the role of *PeXTH* in leaf succulence and salinity tolerance in tobacco plants.

## Materials and methods

### Plant material and salt treatment

One-year-old *P. euphratica* seedlings obtained from the Xinjiang Uygur Autonomous Region of Northwest China were grown in individual pots (10 litres) containing loam soil (1:1 sand to soil) in a greenhouse at Beijing Forestry University. The temperature in the greenhouse was 20–25 °C with a 12h light/12h dark photoperiod and 150 μmol m^–2^ s^–1^ of photosynthetically active radiation. Seedlings were watered with full-strength Hoagland nutrient solution every 2 weeks. A total of 30–40 uniform plants were selected for salt treatment. NaCl treatment started at 50mM and increased stepwise by 50mM weekly, reaching 200mM in the fourth week. Leaves were harvested during the period of increasing NaCl stress, quickly frozen in liquid nitrogen, and stored at –80 °C for real-time quantitative PCR assays.

### Cloning full-length *PeXTH*

Total RNA was extracted from *P. euphratica* leaves using a Plant RNA Kit (QBio Technologies Inc., Beijing, China) according to the manufacturer’s instructions. First-strand cDNA was synthesized by a reverse transcription reaction with 1 μg of total RNA, oligo(dT) primer, and M-MLV reverse transcriptase (Promega, Madison, WI, USA). PCR was performed in a total volume of 25 μl containing 1 μl of the cDNA product, 2 μl of dNTP mixture (2.5mM), 1U of Ex Taq polymerase (Takara, Dalian, China), 2.5 μl of 10× Ex Taq buffer, and 0.5 μl of each primer (10 μM) designed based on the homologous *XTH* sequence of *P. trichocarpa* (NCBI RefSeq acc. XM_002318900). The 5′ to 3′ primer sequences were as follows: forward, GGTCCCACCTCCGAGCTTCC; reverse, GACAGGTAATGCAAGGACG. The PCR product was gel purified and then ligated to the pEASY-T1 vector (TransGen Biotech., Beijing, China) for DNA sequencing.

### Sequence alignment and phylogenetic tree

The full-length amino acid sequences of XTHs were obtained by a similarity search on the NCBI website (http://www.ncbi.nlm.nih.gov/). Signal peptide predictions were performed using the SignalP 4.0 Server online (http://www.cbs.dtu.dk/services/SignalP/). The amino acid sequences were aligned using the ClustalW method in Bioedit Sequence Alignment Editor version 7.0 software. The full-length *PeXTH* cDNA contained an 867bp open reading frame encoding a putative protein of 288 amino acids with an ATG start codon and TAG termination codon (Supplementary Fig. S2 at *JXB* online). The putative amino acid sequence of PeXTH contained three highly conserved regions: (i) the DEIDFEFLG domain, which has been proposed to be the catalytic site of XTH proteins (Campbell and Braam, 1998); (ii) the *N*-glycosylation motif NL(V)SG, which immediately followed the catalytic domain; and (iii) four cysteine residues in the C-terminal portion, which are included in all known XETs (Xu *et al.*, 1995). In addition, PeXTH had a putative N-terminal signal peptide of 24 amino acids (Supplementary Fig. S2).

The phylogenetic tree was generated using the Neighbor–Joining (NJ) method in MEGA version 5.0 software (bootstrap analysis with 1000 replicates) based on the multiple alignments of the amino acid sequences from different plant species in the ClustalW program. The accession numbers of the XTH protein sequences used in multiple sequence alignment and phylogenetic analysis are provided in Supplementary Table S1 at *JXB* online. Phylogenetic analysis revealed that XTHs from different plant species were classified into four subgroups (Supplementary Fig. S3). The putative PeXTH protein from *P. euphratica* was highly homologous to an XTH member from hybrid aspen *Populus tremula* L.×*P. tremuloides* (PttXTH16-17, GenBank accession no. ABM91073; Supplementary Fig. S3).

### Subcellular localization

The full-length *PeXTH* cDNA was cloned into the pMDC85 vector (Curtis and Grossniklaus, 2003) under the control of the *Cauliflower mosaic virus* (CaMV) 35S promoter and fused with the green fluorescent protein (GFP) reporter gene in the 3′ region. A 5′ fusion of GFP was not attempted as the 5′ end of *PeXTH* possesses a signal peptide (Supplementary Fig. S2 at *JXB* online). The fusion of GFP at the 5′ end would cause some artefact in the subcellular localization of the PeXTH protein. Onion epidermal cells were bombarded with 5 μg of the recombinant plasmids with or without the endoplasmic reticulum (ER) marker [cyan fluorescent protein (CFP), ER-ck *CD3-953*; Nelson *et al.*, 2007], as well as the blank vector fused with GFP alone, using a biolistic PDS-1000/He particle delivery system (Bio-Rad, Hercules, CA, USA). After bombardment, the samples were incubated on Murashige and Skoog (MS) solid medium in the dark for 20h at 25 °C and then examined with a Leica SP5 confocal microscope (Leica Microsystems GmbH, Wetzlar, Germany). Plasmolysis was induced by hyperosmotic shock with 400mM sucrose. The confocal settings were excitation at 488nm (GFP) and 453nm (CFP), and emission at wavelengths of 510–535nm (GFP) and 460–485nm (CFP), respectively.

### Expression, purification, and XET activity assay of PeXTH

The full-length *PeXTH* fragment was obtained by PCR amplification using the forward primer 5′-GCGGATCC ATGGCTTCATCGAGTACTGTGCT-3′ (*Bam*HI) and reverse primer 5′-GCGTCGACGGACATGCCGCATTCCGGAG-3′ (*Sal*I). The amplified product was digested with *Bam*HI and *Sal*I, and then ligated into the corresponding site of the pET28a vector (Stratagen, USA). The resulting construct pET28a-*PeXTH* was introduced into *Escherichia coli* BL21. The empty vector pET28a was taken as blank control. Induction was performed by adding 0.5mM IPTG (isopropyl-β-d-thiogalactopyranoside) into the *E. coli* BL21 bacterial culture until the *A*_600_ reached 0.6. Cultures were harvested by centrifugation after 8h of induction and then resuspended in 50mM citrate buffer (pH 5.5). Bacterial cells were lysed by sonication and the crude PeXTH protein (supernatant) was prepared by centrifugation at 12 000rpm for 5min. The supernatant was then purified with Ni-Sepharose media (GE Healthcare, USA) according to the manufacturer’s instruction. Purified PeXTH was dialysed against 2 litres of PBS buffer (pH 7.0). The protein concentration was determined using a Pierce BCA Protein Assay Kit (Thermo, USA). The protein was separated by SDS–PAGE and stained with Coomassie blue R 250.

XET activity was assayed with a colorimetric method developed by Sulová *et al*. (1995). Briefly, 50 μl of purified PeXTH protein was added to a reaction mixture containing 50 μl of tamarind xyloglucan (2mg ml^–1^; Megazyme, Bray, Ireland), and 50 μl of reduced tamarind xyloglucan heptasaccharide (0.5mg ml^–1^; Megazyme). For the pH profile assay, 50 μl of 400mM sodium citrate (pH 3.0–6.0) or sodium phosphate buffer (pH 6.2–8.0) were used in the reaction mixture. The blank controls contain no PeXTH protein. The mixtures were incubated at 30 °C for 30min. Reactions were terminated by adding 100 μl of 1.0M HCl. Afterwards, 800 μl of 20% (w/v) Na_2_SO_4_ and 200 μl of potassium tri-iodide reagent (1% KI and 0.5% I_2_ in water) were added and the mixture was kept in the dark at room temperature for 30min. The optical density was measured at 620nm against the blank. The temperature profile assay was performed in 400mM sodium citrate (pH 6.0) at temperatures ranging from 16 °C to 60 °C. The value for the xyloglucan transglucosylase activity (XET) was expressed in arbitrary units (Sulová *et al.*, 1995) and calculated according to Opazo *et al.* (2010). PeXTH showed a bell-shaped pH profile in XET activity with an optimum at pH 6.0 (Supplementary Fig. S4 at *JXB* online). A rapid decrease in activity was observed in the pH ranges of 4.0–6.0 and 7.0–8.0 (Supplementary Fig. S4). At pH 4.0 and 8.0, the XET activity fell to 14% and 25% of the maximum, respectively (Supplementary Fig. S4). The temperature profile assay showed that the optimum temperature of the enzyme was 37 °C (Supplementary Fig. S4).

### Semi-quantitative RT–PCR and quantitative real-time PCR

The expression level of *PeXTH* in *P. euphratica* and tobacco plants was evaluated by real-time PCR and semi-quantitative reverse transcription–PCR (RT–PCR), respectively, using gene-specific primers (Supplementary Table S2 at *JXB* online). Because *PeXTH* is highly homologous to *NtXTH* (Supplementary Fig. S2), the primers used for semi-quantitative RT–PCR in tobacco plants were designed against different sequences (Supplementary Table S2). In brief, total RNA was isolated from *P. euphratica* (leaves from control and NaCl-treated plants) and tobacco (leaves from transgenic and wild-type plants) using the Plant RNA Kit (QBio Technologies Inc.) according to the manufacturer’s directions. The RNA was treated with RNase-free DNase (Promega), and first-strand cDNA, used as the template of real-time PCR and semi-quantitative RT–PCR, was prepared as described above. The semi-quantitative RT–PCR conditions were: 95 °C for 5min, followed by 26 cycles of 94 °C for 30 s, 55 °C for 30 s, and 72 °C for 30 s, with a final step of 72 °C for 10min. *EF1α* (a housekeeping gene in tobacco; GenBank accession no. D63396) was used as an internal control. The PCR amplification products were separated by 1% (w/v) agarose gel electrophoresis and visualized with ethidium bromide under UV light. The real-time PCR analysis was performed with SYBR Green mix in a Real-Time PCR System (MJ option2, Bio-Rad). Each sample was run in triplicate. *ACT7* (a housekeeping gene in *P. euphtatica*, NCBI RefSeq accession no. XM_002322628) was used as the internal control. The expression data of the target gene, normalized to the expression level of the reference gene (*ACT7*), were analysed using the 2^–ΔΔ^C^_T_^ method (Livak and Schmittgen, 2001). In addition to *PeXTH*, real-time quantitative PCR analysis of the other three *XTH* isoforms (*PeXTH-1*, *PeXTH-3*, and *PeXTH-4*) in control and salinized *P. euphratica* leaves was performed as described above.

In this study, the transcript abundance of two tobacco-intrinsic *NtXTH* genes was examined to determine whether the expression of native *NtXTH* genes was altered by exogenous *PeXTH*. The accession numbers of the two *NtXTH* genes are D86730 and AB017025 (Supplementary Table S2 at *JXB* online). The expression levels were detected by real-time PCR analysis using the primers listed in Supplementary Table S2. *EF1α* was used as the internal control.

### Generation of *35S-PeXTH* tobacco plants

The full-length *PeXTH* cDNA was subcloned into the plant expression vector pGreen0029 (Hellens *et al.*, 2000) under the control of the CaMV 35S promoter to yield recombinant *PeXTH*:pGreen0029 with kanamycin resistance. The *PeXTH*:pGreen0029 construct was introduced into competent *Agrobacterium tumefaciens* cells from strain LBA4404 using the liquid nitrogen freeze–thaw method (An, 1987). Tobacco (*Nicotiana tabacum* cv. Wisconsin 38) plants were infected with *A. tumefaciens* carrying the *PeXTH*:pGreen0029 construct using the leaf disc method (Horsch, 1985). The blank pGreen0029 vector was introduced into wild-type tobacco plants as a control. Putative transgenic plants were selected on MS medium containing 300mg l^–1^ kanamycin and verified by semi-quantitative RT–PCR using *PeXTH*-specific primers (Supplementary Table S2 at *JXB* online). Four independent transgenic lines from the T_2_ generation (L5, L6, L8, and L14) were used for further experiments. Tobacco plants were grown in a growth chamber at 25±1 °C with a light intensity of 50 μmol m^–2^ s^–1^, a photoperiod of 16h light/8h dark, and a relative humidity of 50–60%.

The frequency of kanamycin-resistant plants among T_1_ progeny was examined to determine whether plants harbour a single copy of the transgene. The segregation ratio of kanamycin-resistant (Kan^R^) to kanamycin-sensitive (Kan^S^) seedlings ranges from 2.7 to 3.9, close to the typical Mendelian ratio 3:1 (Supplementary Table S3 at *JXB* online). This suggests that the selected transgenic tobacco lines (L5, L6, L8, and L14) were largely single function insert plants.

### Salt tolerance screening of tobacco plants

Phenotypic screening of tobacco plants under salinity stress was performed using seedlings grown on MS medium and rooted plants acclimated to hydroponics and nursery soil. In brief, seeds from wild-type and transgenic lines (L5, L6, L8, and L14, T_2_ generation) were surface sterilized and germinated in Petri dishes containing half-strength MS medium. The Petri dishes were placed in a growth chamber at 25±1 °C with a photoperiod of 16h light and 8h dark. Seven-day-old seedlings with two leaves were transferred to fresh MS medium supplemented with 0mM or 150mM NaCl. Root length and survival rates were measured after 10 d of salt stress; seedlings with new green leaves were considered surviving plants.

Short-term and long-term hydroponic treatments were carried out in this study. For the short-term study, 4-week-old rooted plants of the wild type, vector control, and selected transgenic tobacco lines (L5 and L14) grown on MS medium were transferred to one-quarter strength Hoagland nutrient solution. After 7 d of acclimation, the plants were treated with 0 or 150mM NaCl for 7 d. The nutrient solution was refreshed every 2 d during the period of cultivation. Air temperature in the greenhouse was 25±1 °C. Light intensity was 150–200 μmol m^–2^ s^–1^, with a photoperiod of 16h light/8h dark. At the end of the salt treatment, leaves and roots of tobacco plants were harvested to measure leaf fresh weight (FW) and root length. The long-term salt treatment was performed at the Institute of Forest Botany and Tree Physiology, Göttingen University (Germany). Rooted plants of wild-type and transgenic lines were subjected to 80 d of increasing salt stress. The NaCl concentration was increased weekly from 50mM to 200mM, and then was kept at 200mM until the end of the experiment. Control plants were treated without the addition of NaCl. Plant roots were continuously aerated by passing air through the solution. The temperature in the greenhouse chamber was 24±1 °C with a 16h photoperiod (7:00–23:00h) and 150 μmol photons m^–2^ s^–1^ of photosynthetically active radiation. Wild-type and transgenic plants were harvested, and all sampled materials (root, leaf, and stem) were oven-dried (65 °C for 3 d) to obtain the dry mass.

In addition to hydroponic cultures, wild-type control and transgenic plants were grown in nursery soil for salt treatment. In brief, 1-week-old seedlings of the wild type, vector control, and the two transgenic lines (L5 and L14) germinated on half-strength MS medium were grown in nursery soil for a further 30 d. Salt treatment was applied by top watering with 150mM NaCl. After 7 d of salt stress, tobacco leaves were harvested and the FW was assessed. The soil cultivation was performed in a greenhouse at 25±1 °C, with a light intensity of 100–150 μmol m^–2^ s^–1^ and a photoperiod of 16h light/8h dark.

### Leaf photosynthesis, chlorophyll *a* fluorescence, and succulence analysis

Four-week-old rooted transgenic and wild-type tobacco plants were transferred from MS solid medium to one-quarter strength Hoagland nutrient solution. After 7 d of acclimation, the plants were transferred to fresh solution supplemented with 0, 100, or 150mM NaCl. In this study, the third–fifth mature leaves from the tip were used for photosynthesis, chlorophyll *a* fluorescence, and succulence measurements. The net photosynthetic rate was measured using a LI-6400 photosynthesis system (Li-Cor Inc., Lincoln, NE, USA) after 7 d of salt treatment. Chlorophyll *a* fluorescence of 0.5h dark-adapted leaves was measured with an Imaging-PAM chlorophyll fluorometer (Walz, Effeltrich, Germany). Maximum photochemical efficiency of photosystem II (PSII; *F*_v_/*F*_m_) was calculated as previously described (Wang *et al.*, 2007). After the gas exchange and fluorescence measurements, the leaves were harvested for succulence, water-retaining capacity (WRC), anatomy, and tissue ion analysis. For succulence measurement, the FW was determined immediately and the leaf area (A) quantified using a scanner (ScanJet 4C/T, Hewlett Packard). The dry weight (DW) was obtained after the samples were oven-dried at 65 °C for 3 d. The leaf succulence degree (LSD), as indicated by the ratio of FW to DW (Zotz and Winter, 1994) and leaf water content per unit area (Mantovani, 1999), was calculated as follows:


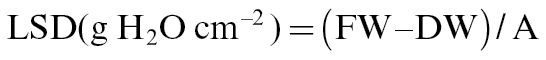


### Leaf water-retaining capacity

Leaf WRC was examined to compare the difference in maintaining water status between wild-type and transgenic plants. Immediately after the upper mature leaves (the third–fifth from the tip) were excised from control and salt-treated plants (150mM), the FW (FW_0_) was obtained. Thereafter, leaf samples were placed on laboratory bench under a light intensity of 150–200 μmol m^–2^ s^–1^, and water loss from the leaf surface was regularly measured during the period of 120min air exposure. Air temperature was 25 °C and relative humidity was 40%. The WRC was calculated as follows:


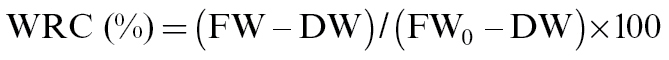


where FW is the leaf fresh weight during the period of air exposure, and DW represents the dry weight.

### Leaf anatomy

Sample preparation for the evaluation of leaf anatomy followed that of Chen *et al.* (2009). In brief, the third–fifth mature leaves from the tip were sampled from transgenic and wild-type tobacco plants in the presence and absence of NaCl stress (150mM, 7 d), and then fixed in FAE (2% formaldehyde, 5% acetic acid, and 63% ethanol). There was no additional osmoticum included in the fixation solution of salt-stressed plants, since FAE solution is typically used to stabilize cell structures of specimens sampled from control and NaCl treatments (Ottow *et al.*, 2005; Luo *et al.*, 2009). These leaf samples were transferred to 70% (v/v) ethanol before dehydration. Samples were subjected to dehydration by subsequent incubation in the following solutions: 70% ethanol:acetone (1:1) for 2h, 70% ethanol:acetone (1:3) for 2h, 100% acetone for 2h, and then 100% acetone again for 5h. Thereafter, samples were transferred to water-free acetone (twice for 24h). Infiltration with plastic was carried out by the following steps: water-free acetone:plastic (1:1) for 24h, water-free acetone:plastic (1:3) for 24h, 100% plastic for 24h (twice). The plastic was a 1:1 mixture of styrene (Merck-Schuchardt) and butyl methacrylate (Sigma-Aldrich) containing 1% benzoyl peroxide stabilized with 50% phthalate (Fritz, 1989). Polymerized samples were cut into 1 μm thick sections using an ultramicrotome. Cuttings were stained with toluidine blue and mounted on gelatin-coated glass slides. Digital images of the prepared slides were obtained using a digital camera (Nikon CoolPix 990, Nikon) attached to a light microscope (Axioskop, Zeiss). Anatomical features, such as leaf thickness, perimeter, and area per cell, were measured using ImageJ software (version 1.46).

### Leaf ion analysis

The third–fifth mature leaves from the tip were harvested for ion analysis. Dried leaf samples (0.2g) were ground into a fine powder to pass through a 1mm sieve. After digestion with 1M HNO_3_, the Na^+^ content was determined using a Perkin-Elmer 2280 atomic absorption spectrophotometer (PerkinElmer, Inc., Wellesley Hills, MA, USA) and the Cl^–^ content was measured as described by Chen *et al.* (2001). Ion concentrations were expressed as the ion content based on the volume of leaf water.

### Localization of Na^+^ in leaf cells

Ten-day-old transgenic and wild-type tobacco seedlings were transferred to MS medium supplemented with 0mM or 150mM NaCl. After 7 d of salt treatment, seedlings were incubated with the sodium-specific dye CoroNa-Green AM (Invitrogen, Carlsbad, CA, USA) for 12h. The stained seedlings were washed with distilled water three times. The specific fluorescence in leaves was visualized under a Leica SP5 confocal microscope (Leica Microsystems GmbH, Wetzlar, Germany) with excitation at 488nm and emission at 500–530nm. Three-dimensional (3D) reconstructed images (maximum) of mesophyll cells were used to calculate the relative fluorescence intensity using Image-Pro Plus version 6.0 software.

### Statistical analysis

All experiments were repeated at least three times. The data were subjected to analysis of variance (ANOVA). Significant differences between means were determined by Duncan’s multiple range test. Unless otherwise stated, *P*<0.05 was considered significant.

## Results

### Subcellular localization of PeXTH

**Fig. 1.**
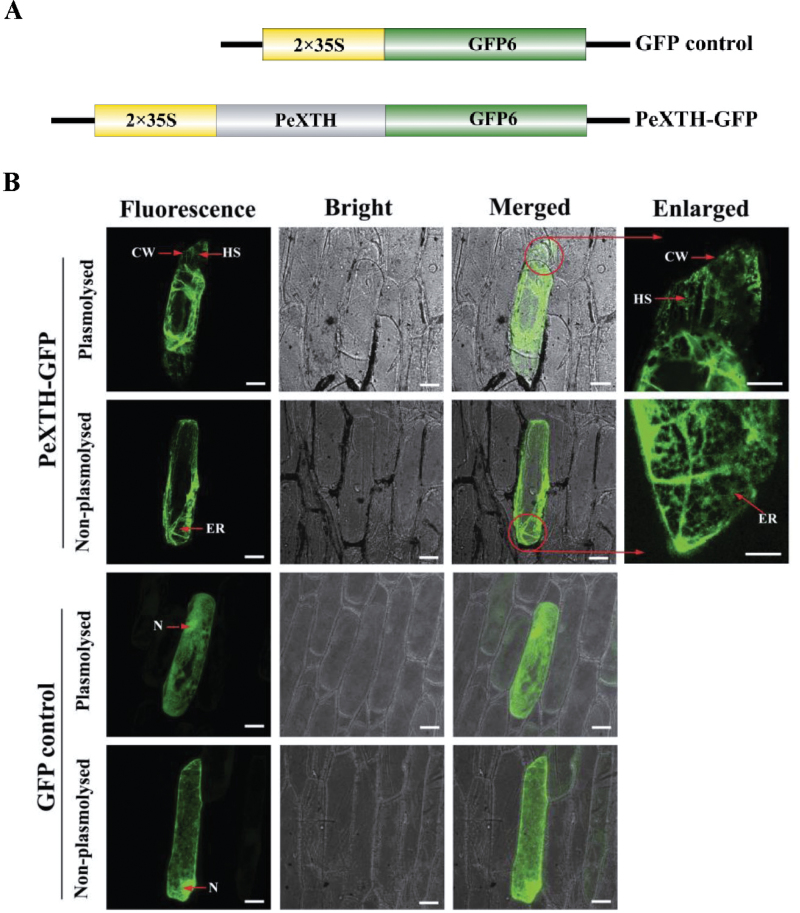
Subcellular localization of PeXTH by transient transformation in onion epidermal cells. (A) Diagram of the *PeXTH*–*GFP* fusion construct and *GFP* control. (B) Representative images of *PeXTH*-transgenic onion cells and *GFP* control (plasmolysed and non-plasmolysed). Plasmolysis was induced by hyperosmotic shock with 400mM sucrose. Circled structures were enlarged to show Hechtian strands and endoplasmic reticulum in plasmolysed and non-plasmolysed onion cells. CW, cell wall; HS, Hechtian strands; N, nucleus; ER, endoplasmic reticulum. Scale bar=50 μm. (This figure is available in colour at *JXB* online.)

PeXTH–GFP was constructed to transform onion epidermal cells and incubated for 20h (Fig. 1A). Green fluorescence was observed in both non-plasmolysed cells and plasmolysed cells (plasmolysis was induced by hyperosmotic shock with 400mM sucrose; Fig. 1B). The PeXTH–GFP fusion protein was present in structures similar to the ER network in non-plasmolysed cells (Fig. 1B; Supplementary Fig. S5 at *JXB* online). Interestingly, the green fluorescence detected in plasmolysed cells expanded to the cell walls and Hechtian strands, which probably contain plasma membrane and ER (Fig. 1B). These results indicate that PeXTH was produced in the ER and transported to the cell wall, possibly exclusively through the secretory pathway. Cells transformed with GFP alone showed GFP fluorescence throughout the cytoplasm and nucleus in both plasmolysed and non-plasmolysed cells (Fig. 1B).

### Root and leaf growth, survival, and salinity tolerance

**Fig. 2.**
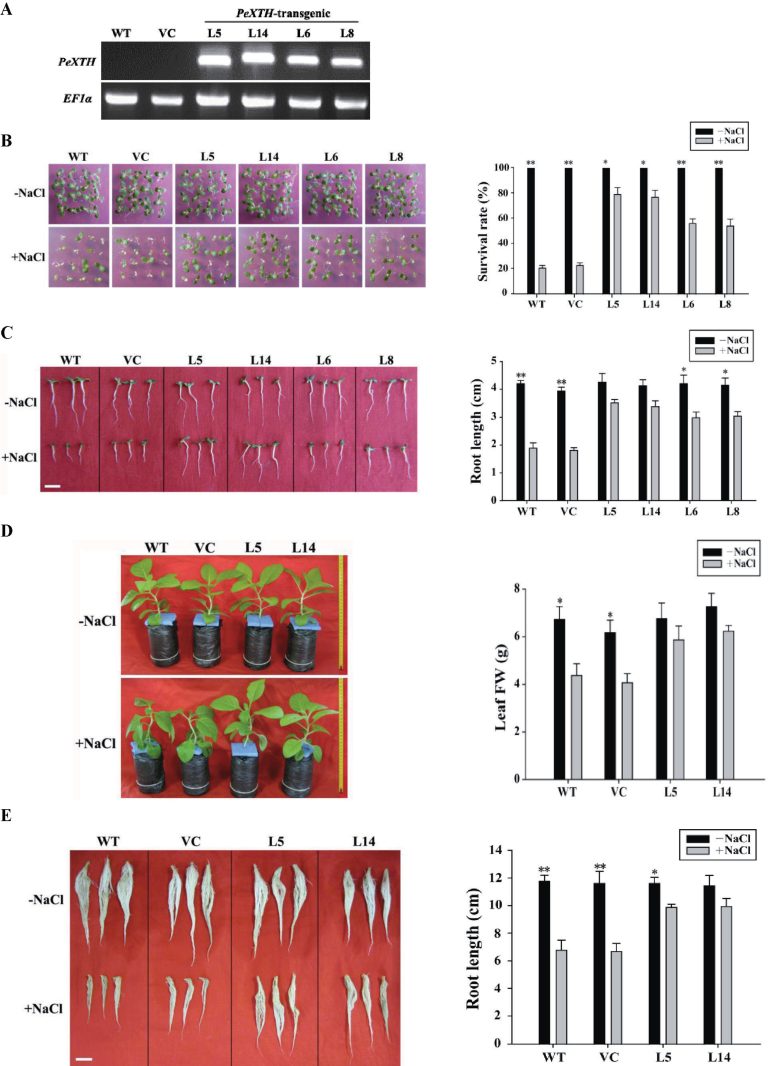
Salt tolerance of wild-type (WT) tobacco, vector control (VC), and *PeXTH*-transgenic plants. (A) Semi-quantitative RT–PCR analysis. *EF1α* was used as the internal control. (B and C) Salt tolerance test on MS medium. Seeds from wild-type and transgenic lines (L5, L6, L8, and L14, T_2_ generation) were allowed to germinate on half-strength MS medium and then transferred to MS medium supplemented with 150mM NaCl. Control plants were grown on MS medium without the addition of NaCl. Representative images show the plant survival rate (B) and root length (C) after 10 d of salt stress. (D and E) Salt tolerance test in hydroponic culture. Four-week-old plants were acclimated to one-quarter strength Hoagland nutrient solution for 7 d, and then salinized with 0 or 150mM NaCl. Representative images show the leaf fresh weight (FW) (D) and root length (E) after 7 d of salt stress. Scale bar=2.0cm. Each column is the mean of three independent experiments. The bars represent the standard error of the mean. *P<0.05, **P<0.01 control versus salt treatment. (This figure is available in colour at *JXB* online.)

Salt stress up-regulated *PeXTH* transcription in *P. euphratica* leaves (Supplementary Fig. S1 at *JXB* online), implying that salt-induced *PeXTH* expression may contribute to salinity tolerance in this woody species. In an attempt to evaluate a functional role for *PeXTH* in salinity tolerance, *PeXTH* was transferred to tobacco under the control of the CaMV 35S promoter. A remarkable *PeXTH* transcript level was detected in four independent transgenic lines (designated L5, L6, L8, and L14; Fig. 2A). Four series of salt treatments were carried out to determine the salinity tolerance of transgenic plants at different stages of development. (i) MS agar medium. Young seedlings of the wild type, vector control, and transgenic lines (L5, L6, L8, and L14) were grown in MS medium supplemented with 0 or 150mM NaCl. After 10 d of salt stress, wild-type and vector control seedlings exhibited symptoms of salt injury (e.g. leaves turned yellow and root growth was suppressed by 62–64%; Fig. 2B, C). Compared with the wild type and vector control, *PeXTH*-transgenic seedlings, especially L5 and L14, exhibited a greater capacity to tolerate the salinity stress. Leaves were not severely injured in salinized transgenic seedlings and root length was reduced less by salt treatment (Fig. 2B, C). After salt treatment, the transgenic lines exhibited a significantly higher survival rate than the wild type and vector control (79% for L5 versus 21–23%; Fig. 2B). Under no-salt conditions, the transgenic lines did not significantly differ from the wild type and vector control in regards to root and shoot growth (Fig. 2B, C). (ii) Short-term hydroponic culture. Tobacco plants grown on MS medium were transferred to hydroponic culture and salinized with 150mM NaCl. Salt-stressed plants of the wild type and vector control displayed wilted leaves, whereas the water shortage was not seen in transgenic lines (Fig. 2D). Moreover, the two *PeXTH*-overexpressing lines (L5 and L14) exhibited a less reduced root length and FW of leaves, compared with the wild type and vector control (Fig. 2D, E). (iii) Long-term hydroponic culture. Wild-type and transgenic plants were subjected to 80 d of increasing salt stress (NaCl saline increased weekly from 50mM to 200mM, and then kept at 200mM until the end of the experiment). Plants were harvested before flowering. The data show that transgenic plants had a greater biomass than the wild type, although the dry weights of leaves, roots, and stem were reduced by the salt exposure (Supplementary Fig. S6A at *JXB* online). (iv) Soil culture. Transgenic plants grown in nursery soil exhibited a higher leaf FW than the wild type and vector control under saline conditions (Supplementary Fig. S6B, C). This is consistent with the observations from MS media and hydroponic cultures (Fig. 2, Supplementary Fig. S6B, C). Collectively, the results show that *PeXTH*-transgenic plants exhibited a greater capacity to tolerate salinity than the wild type and vector controls irrespective of culture media, for example MS medium, hydroponic solution, and nursery soil.

In this study, the transcript abundance of two tobacco-intrinsic *NtXTH* genes was examined to determine whether the expression of native *NtXTH* genes was altered by exogenous *PeXTH*. *NtXTH* genes exhibited a higher expression (10–23%) in salt-treated plants, as compared with no-salt controls (Supplementary Fig. S7 at *JXB* online). In general, the tobacco-intrinsic *NtXTH* genes were not significantly altered by the exogenous *PeXTH* gene irrespective of control and salt treatment (Supplementary Fig. S7).

### Leaf anatomy

**Fig. 3.**
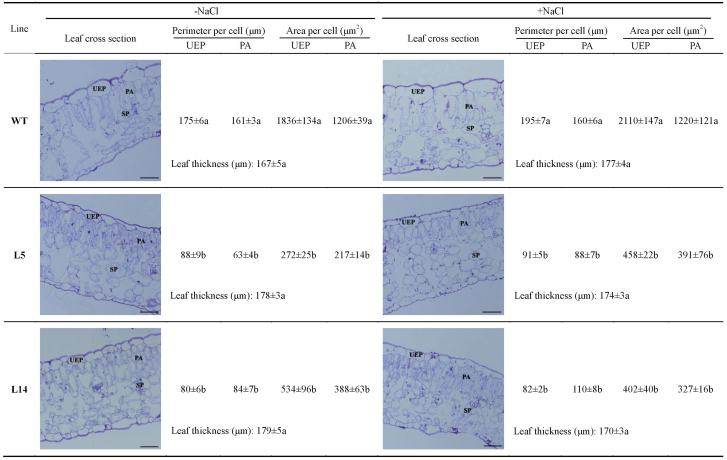
Leaf anatomy of wild-type (WT) tobacco and *PeXTH*-transgenic (L5 and L14) plants under control and saline conditions (150mM NaCl). Embedded leaf samples were cut into 1 μm thick sections and stained with toluidine blue, then mounted on gelatin-coated glass slides. Digital images were obtained with a digital camera (Nikon CoolPix 990, Nikon) attached to a light microscope (Axioskop, Zeiss). Each value (±SE) is the mean of six leaves from three individual plants. Values labelled with different letters in the same column are significantly different between wild-type and transgenic lines at *P*<0.05. UEP, upper epidermis; PA, palisade parenchyma; SP, spongy parenchyma. Scale bar=40 μm. (This figure is available in colour at *JXB* online.)

Leaf anatomy was examined using cross-sections from transgenic and wild-type tobacco plants. Overexpression of *PeXTH* in tobacco did not significantly increase leaf thickness, but it altered leaf anatomy (Fig. 3). Wild-type tobacco displayed typical, larger upper epidermal (UEP) cells, whereas smaller UEP cells were observed in the L5 and L14 transgenic lines (Fig. 3). Similarly, the transgenic lines exhibited smaller palisade parenchyma (PA) cells compared with the wild-type cells (Fig. 3). Notably, two to three layers of PA cells were found in L5 and L14, compared with one layer of PA cells in the wild-type cells (Fig. 3). Moreover, in the transgenic lines, there were fewer intercellular air spaces between spongy mesophyll cells, particularly for L14 (Fig. 3). However, the transgenic leaves did not differ from wild-type leaves in the lower epidermis (Fig. 3). In wild-type and transgenic lines, leaf thickness and anatomy were not altered significantly by salt treatment (Fig. 3).

**Fig. 4.**
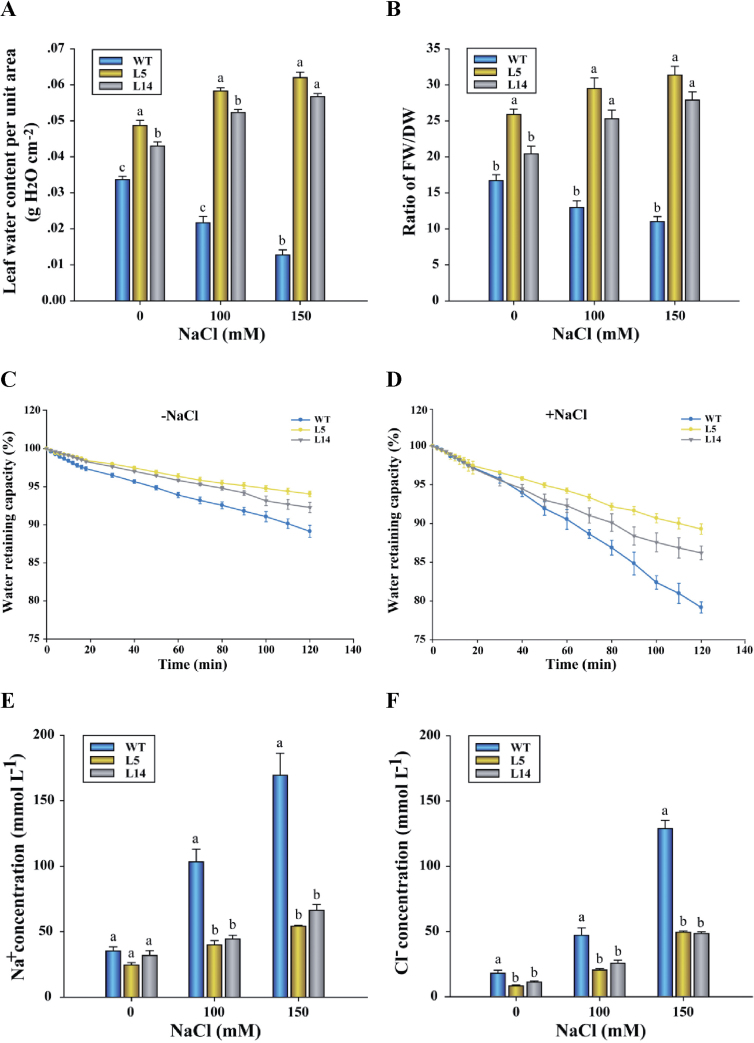
Leaf succulence, water-retaining capacity (WRC), and Na^+^ and Cl^–^ concentrations in wild-type (WT) tobacco and *PeXTH*-transgenic (L5 and L14) plants. WT and *PeXTH*-transgenic plants were cultivated in one-quarter strength Hoagland nutrient solution supplemented with 0, 100, or 150mM NaCl. After 7 d of salt treatment, the leaf water content per unit area, ratio of fresh weight to dry weight, WRC, and Na^+^ and Cl^–^ concentrations were measured in WT and transgenic plants. (A and B) Leaf succulence. Leaf succulence was indicated by water content per unit area (A), and ratio of fresh weight to dry weight (B). (C and D) Leaf WRC. The WRC represents the percentage of total water retained in leaves during the period of air exposure (see the Materials and methods). Each point is the mean of three plants. (E and F) Leaf salt concentrations. Na^+^ and Cl^–^ concentrations were expressed as the ion content based on the volume of leaf water. In A, B, E, F, each column is the mean of three independent experiments. The bars represent the standard error of the mean. Columns labelled with different letters, a, b, and c, indicate significant differences between wild-type and transgenic lines at *P*<0.05 under control and salt treatment.

Leaf water content per unit area (WC/A) and the FW/DW ratio were measured as an indication of leaf succulence (Zotz and Winter, 1994; Mantovani, 1999). Transgenic lines L5 and L14 exhibited 36% higher WC/A and 39% higher FW/DW than the wild-type plants under no-salt conditions (Fig. 4A, B). The same trend was seen after exposure to NaCl (100–150mM; Fig. 4A, B). Interestingly, transgenic plants tended to increase leaf succulence upon salt stress, whereas a remarkable decrease was measured in the wild-type plants (Fig. 4A, B).

Leaf WRC of wild-type and transgenic plants was compared in this study. The WRC represents the percentage of total water retained in leaves during the period of air exposure. Salinized plants showed a more rapid water loss (10–20%) than no-salt controls (5–10%) after 2h of air exposure (Fig. 4C, D). Of note, *PeXTH*-transgenic lines L5 and L14 exhibited typically higher WRC than the wild-type plants, regardless of control and salt treatment (Fig. 4C, D). The data show that highly packed leaf cells in transgenic plants lowered water loss during the period of air exposure. Similarly, detached leaves from *CaXTH3*-transgenic tomato displayed a lower transpirational water loss than wild-type plants (Choi *et al.*, 2011).

### Leaf Na^+^ and Cl^–^ concentrations

**Fig. 5.**
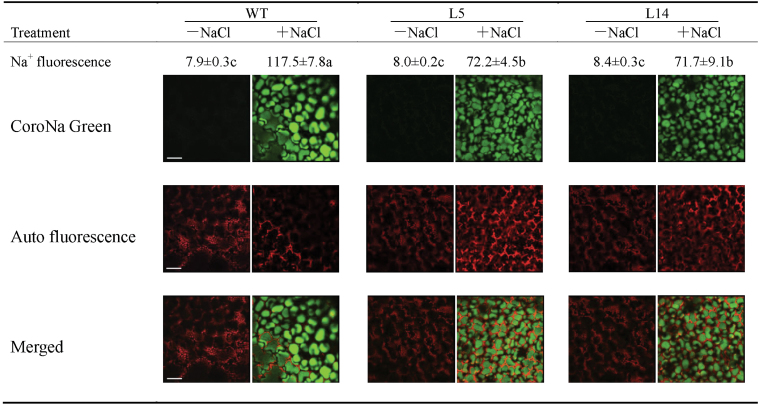
Na^+^ levels in the mesophyll cells of wild-type (WT) tobacco and *PeXTH*-transgenic (L5 and L14) plants. Ten-day-old seedlings were transferred to MS medium supplemented with 0 or 150mM NaCl. After 7 d of salt treatment, seedlings were incubated with CoroNa-Green AM (green, sodium-specific) for 12h. The autofluorescence of chlorophyll is also shown. The mean fluorescence values labelled with different letters, a, b, and c, are significantly different between wild-type and transgenic lines at *P*<0.05. Scale bar = 20 µm. (This figure is available in colour at *JXB* online.)

In transgenic and wild-type tobacco plants, leaf Na^+^ and Cl^–^ concentrations increased with increasing amounts of NaCl in the nutrient solution (Fig. 4E, F). However, a more pronounced increase was observed in wild-type plants (Fig. 4E, F). In the presence of 150mM NaCl, the mean Na^+^ and Cl^–^ concentrations reached 169 mmol l^–1^ and 128 mmol l^–1^, respectively, in the wild-type leaves, which were 1.5- to 2-fold higher concentrations than those measured in transgenic leaves (Fig. 4E, F). Sodium accumulation in mesophyll cells was examined using sodium-specific dye. Compared with the transgenic plants, wild-type plants exhibited larger individual mesophyll cells, which were surrounded by autofluorescence from chloroplasts (Fig. 5). Imaging profiles showed that Na^+^-specific fluorescence occurred primarily in vacuolar regions (Fig. 5). Notably, the fluorescence intensity of the two transgenic lines was 39% less than the fluorescence observed in wild-type plants (Fig. 5). Under no-salt stress, CoroNa-Green fluorescence was almost undetectable in the mesophyll cells of transgenic and wild-type leaves (Fig. 5), due to low Na^+^ content in the mesophyll cells.

### Photosynthesis and chlorophyll *a* fluorescence

**Fig. 6.**
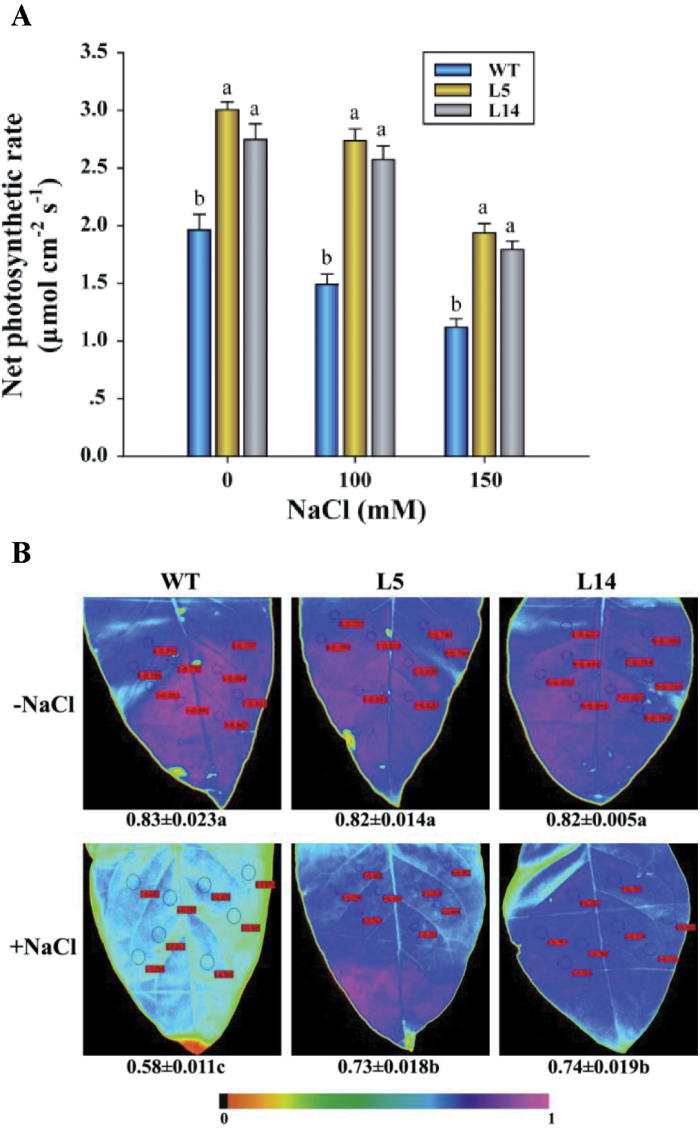
Net photosynthetic rate (Pn) and the maximum photochemical efficiency (*F*_v_/*F*_m_) of wild-type (WT) tobacco and *PeXTH*-transgenic (L5 and L14) plants. Wild-type (WT) tobacco and transgenic plants were cultivated in one-quarter strength Hoagland nutrient solution supplemented with 0, 100, or 150mM NaCl. After 7 d of salt treatment, Pn and chlorophyll *a* fluorescence were measured using upper mature leaves (third–fifth from the tip) of WT and transgenic plants. (A) The net photosynthetic rate. Each column is the mean of three independent experiments. The bars represent the standard error of the mean. Columns labelled with different letters, a and b, indicate significant differences between wild-type and transgenic lines at *P*<0.05 under control and salt treatment. (B) Representative fluorescence images showing the maximum photochemical efficiency (*F*_v_/*F*_m_) of WT and transgenic plants treated with 0 or 150mM NaCl. The sampling points are indicated by circles, and *F*_v_/*F*_m_ values are shown adjacent to the measuring points. Each value is the mean of four plants, and values (±SE) labelled with different letters, a, b, and c, are significantly different at *P*<0.05 between control and salt treatment. (This figure is available in colour at *JXB* online.)

Photosynthetic capacity, indicated by the net photosynthetic rate (Pn) and the maximum photochemical efficiency (*F*_v_/*F*_m_), was decreased in response to NaCl in wild-type and transgenic plants (Fig. 6). However, Pn was significantly higher in the transgenic lines than in the wild-type under both control and saline conditions (Fig. 6A). *F*_v_/*F*_m_ of the wild-type leaves decreased significantly from 0.83 to 0.58 after 7 d of NaCl treatment (150mM), while in transgenic leaves *F*_v_/*F*_m_ was less reduced (Fig. 6B).

## Discussion

XTHs can modify and reorganize the cellulose–xyloglucan framework by catalysing cleavage and re-ligation of the xyloglucan chains in the plant cell wall (Fry *et al.*, 1992; Nishitani and Tominaga, 1992; Rose *et al.*, 2002; Vissenberg *et al.*, 2005). Via its XET function, XTH can cause alterations in xyloglucan–cellulose linkages and regulate cell wall extensibility (Vissenberg *et al.*, 2005; Miedes *et al.*, 2011). XTHs contribute to the cell elongation process in a number of species, including *Festuca pratensis* (*FpXET1*; Reidy *et al.*, 2001), *Sagittaria pygmaea* (*SpXTH1*; Ookawara *et al.*, 2005), and *Arabidopsis thaliana* (*AtXTH18* and *AtXTH21*; Osato *et al.*, 2006; Y.B. Liu *et al.*, 2007). The homology of PeXTH to XTH members from other poplars and the conserved DEIDFEFLG motif and putative residues for *N*-glycosylation (Campbell and Braam, 1998) identified in PeXTH (Supplementary Figs S2, S3 at *JXB* online) indicate that this enzyme possesses structure and activity similar to those of other XTH proteins. The enzymatic analysis demonstrated that XET activity of PeXTH protein depends on pH and temperature. PeXTH exhibited optimum XET activity at 37 °C with an acidic pH of 6.0 (Supplementary Fig. S4). Localization of PeXTH in the ER and cell wall (Fig. 1; Supplementary Fig. S5) agrees with the previous study by Genovesi *et al.* (2008), who found that ZmXTH1 is localized in the cell wall and causes alterations in the cell wall composition.

In the present study, overexpression of *PeXTH* increased salt tolerance in transgenic tobacco plants, irrespective of development stage and culture medium (Fig. 2; Supplementary Fig. S6 at *JXB* online). Similarly, *CaXTH3* overexpression improved salt tolerance in *Arabidopsis* and tomato plants (Cho *et al.*, 2006; Choi *et al.*, 2011). Three homologous XTH genes found in hot pepper, *CaXTH1*, *CaXTH2*, and *CaXTH3*, were up-regulated in response to salt stress (Cho *et al.*, 2006). CaXTH3 is likely to be involved in cell wall remodeling to strengthen the wall layers and thus participates in the protection of mesophyll cells against water deficit and high salinity (Cho *et al.*, 2006). Ectopic expression of *CaXTH3* in *Arabidopsis* caused highly populated small-sized cells in transgenic leaves (Cho *et al.*, 2006). Similar alterations in anatomy were observed in *PeXTH*-transgenic tobacco (Fig. 3). It is assumed that the constitutive presence of CaXTH3 enhanced cell wall biogenesis, which, in turn, results in the formation of numerous small-sized cells in leaves (Cho *et al.*, 2006). In contrast to *Arabidopsis*, *CaXTH3*-transgenic tomato did not alter leaf morphology during early development of the leaves (Choi *et al.*, 2011). Detached leaves from *CaXTH3-*transgenic plants exhibited a lower water loss than the wild type (Choi *et al.*, 2011). It is suggested that increased cell wall remodelling activity of CaXTH3 in guard cells reduces transpirational water loss in response to dehydration stress (Choi *et al.*, 2011). Similarly, *PeXTH*-transgenic tobacco displayed a high WRC (Fig. 4). In salt-resistant maize, expression of ZmXET1 transcript is elevated under saline conditions (Geilfus *et al.*, 2011). Cauliflower florets exhibited significantly higher XET activity after being treated with 300mM NaCl (Takeda and Fry, 2004). Accordingly, NaCl treatment induced 2.5- to 3.2-fold higher expression of *PeXTH* in *P. euphratica* leaves (Supplementary Fig. S1 at *JXB* online). Ottow *et al.* (2005) found that *P. euphratica* developed succulent leaves after prolonged exposure to salt stress. Therefore, PeXTH can be inferred to contribute to salinity tolerance through anatomical modifications.

*PeXTH* overexpression increased leaf succulence in this study (Fig. 4). Of note, the succulence was accompanied by highly packed palisade parenchyma cells but was not due to greater leaf thickness (Fig. 3). This is different from the finding in the salt-resistant *P. euphratica*, which displayed thicker and swollen leaves under NaCl treatment (Ottow *et al.*, 2005). However, the increased numbers of cell layers, tightly packed cells in combination with a reduction of intercellular spaces in transgenic tobacco (Fig. 3), were also observed in *P. euphratica* succulent leaves (Ottow *et al.*, 2005). In the present study, *PeXTH*-transgenic plants exhibited an increased number of mesophyll cells, though they were smaller in size. This finding is in agreement with findings in *CaXTH3*-transgenic *Arabidopsis* leaves (Cho *et al.*, 2006). However, both leaf epidermal cells and mesophyll cells were found to be much larger in *BcXTH1*-transgenic *Arabidopsis* plants than in wild-type plants (Shin *et al.*, 2006). These contrasting morphological alterations are presumably due to species differences in XTH activity.

In transgenic tobacco plants, the increased succulence diluted the toxic Na^+^ and Cl^–^ concentrations at the tissue and cellular levels. The low salt concentrations were due mainly to sufficient water being retained in the succulent leaves (Fig. 4). Highly packed palisade parenchyma cells and reduced air gaps between mesophyll cells probably resulted in high WRC in *PeXTH*-transgenic tobacco (Fig. 4). This is favourable for salinized plants to slow the increase of Na^+^ and Cl^–^ in leaves (Figs 4, 5). In contrast, wild-type plants exhibited wilted leaves with an evident decline of water storage per unit of leaf area or DW after being subjected to salt stress (Figs 2, 4). This is in part due to the typically lower leaf WRC (Fig. 4), in addition to the reduced water uptake in roots under salt stress. Leaf gas exchange was decreased in response to NaCl (Fig. 6), resulting from the salt-induced stomatal closure in tobacco plants. A decline of stomatal opening is helpful for plants to maintain the water status during the period of salt stress (Chen *et al.*, 2002*a*, *b*, 2003). However, the wild-type plants were not able to retain the water status under salt stress, since water loss from the leaf surface was continuing even though the stomata were closed in salinized plants (Fig. 4). As a result, the decline in leaf water status led to a marked elevation of salt concentrations in the leaf tissues and mesophyll cells (Figs 4, 5). However, the differences in accumulation of Na^+^/Cl^–^ between the wild type and L5/14 lines in response to NaCl cannot be explained through differences in FW/DW ratios since the latter differs much less between the lines (Fig. 4). At present, the possibility cannot be excluded that uptake of Na^+^/Cl^–^ differs significantly between wild-type and L5/14 plants. It is likely that transgenic plants had a greater capacity to restrict Na^+^/Cl^–^, compared with the wild type. The constitutive expression of *PeXTH* in tobacco may also have influenced root anatomy and thereby, reduced the salt transport to leaves.

Compared with wild-type plants, *PeXTH*-transgenic leaves exhibited a higher capacity to maintain the net photosynthetic rate and the maximum photochemical efficiency under salt stress (Fig. 6). This is partly due to the increased number of palisade parenchyma cells, which exerted dilution effects reducing the salt toxicity to the photosynthetic apparatus and CO_2_ assimilation. Moreover, the reduced intercellular air space is, probably, favourable to improving carbon economy in photosynthetically active mesophyll cells as *PeXTH*-transgenic plants exhibited 47–78% greater net photosynthetic rates under control and salt treatments (100–150mM; Fig. 6).

In conclusion, overexpression of *PeXTH* enhanced salt tolerance in tobacco plants by the development of leaf succulence. Highly packed palisade parenchyma cells in *PeXTH*-transgenic plants led to high water storage per unit of leaf area or DW, diluting the salt concentrations in succulent leaves under NaCl stress. Moreover, the anatomical alterations caused by *PeXTH* overexpression increased leaf WRC, which lowered the increase in leaf salt concentrations under salinity stress. In addition, the increased number of mesophyll cells and reduced intercellular air space improved carbon economy and photosynthesis. Taken together, these anatomical and physiological modifications are beneficial for *PeXTH*-transgenic plants dealing with salinity stress.

## Supplementary data

Supplementary data are available at *JXB* online.

Figure S1. Expression profiles of *XTH* isoforms in *P. euphratica* leaves under salt stress.

Figure S2. Multiple sequence alignment of PeXTH (xyloglucan endotransglucosylase/hydrolase from *P. euphratica*) with other XTHs from different species.

Figure S3. Phylogenetic relationships between PeXTH and other representative XTH proteins from different plant species.

Figure S4. Effects of pH (A) and temperature (B) on xyloglucan endotransglucosylase (XET) activity of purified PeXTH protein.

Figure S5. Co-localization of PeXTH–GFP with the endoplasmic reticulum marker (ER-ck *CD3-953*) in non-plasmolysed onion cells.

Figure S6. Salt tolerance of wild-type tobacco and *PeXTH*-transgenic plants grown in hydroponics and nursery soil supplemented or not with NaCl.

Figure S7. Expression of *PeXTH* gene and two tobacco-intrinsic *NtXTH* genes in wild-type (WT), vector control (VC), and *PeXTH*-transgenic tobacco plants (L5 and L14).

Table S1. Accession numbers of XTH protein sequences used in multiple sequence alignment and phylogenetic analysis.

Table S2. Primers used for quantitative real-time PCR.

Table S3. The segregation ratio of kanamycin-resistant (Kan^R^) to kanamycin-sensitive (Kan^S^) seedlings among T_1_ progeny of *PeXTH*-transgenic plants.

Supplementary Data
