# Supplementary material for: Populus euphratica XTH overexpression enhances salinity tolerance by the development of leaf succulence in transgenic tobacco plants
Source: J Exp Bot. 2013 Oct 1;64(14):4225–38. doi: 10.1093/jxb/ert229 (PMC3808310; doi:10.1093/jxb/ert229)
Supplement: Supplementary Data [file supp_64_14_4225__index.html]

 Populus euphratica XTH overexpression enhances salinity tolerance by the development of leaf succulence in transgenic tobacco plants — Supplementary Data 

# *Populus euphratica* XTH overexpression enhances salinity tolerance by the development of leaf succulence in transgenic tobacco plants

## Supplementary Data

Data files

**Files in this Data Supplement:**

- Supplementary Data - Supplementary Data
