# Supplementary material for: Populus euphratica XTH overexpression enhances salinity tolerance by the development of leaf succulence in transgenic tobacco plants
Source: J Exp Bot. 2013 Oct 1;64(14):4225–38. doi: 10.1093/jxb/ert229 (PMC3808310; doi:10.1093/jxb/ert229)
Supplement: Supplementary Data [file supp_ert229_ert229_Supplementary_data_corrected.pdf]

***Populus euphratica* XTH overexpression enhances salinity tolerance by the development of leaf succulence in transgenic tobacco plants**

Yansha Han, Wei Wang, Jian Sun, Mingquan Ding, Rui Zhao, Shurong Deng, Feifei Wang, Yue Hu, Yang Wang, Yanjun Lu, Liping Du, Zanmin Hu, Heike Diekmann, Xin Shen, Andrea Polle, Shaoliang Chen

Supplementary Table S1. Accession numbers of XTH protein sequences used in multiple sequence alignment and phylogenetic analysis.

Sequence name	Accession number	Sequence name	Accession number
AtXTH1	NP_193044	AtXTH2	NP_193045
AtXTH3	NP_189141	AtXTH4	NP_178708
AtXTH5	NP_196891	AtXTH6	NP_569019
AtXTH7	NP_195494	AtXTH8	NP_563892
AtXTH9	NP_192230	AtXTH12	NP_200561
AtXTH13	NP_200562	AtXTH14	NP_194312
AtXTH15	NP_193149	AtXTH16	NP_566738
AtXTH17	NP_176710	AtXTH18	NP_194757
AtXTH19	NP_194758	AtXTH20	NP_199618
AtXTH21	NP_179470	AtXTH24	NP_194756
AtXTH27	NP_178294	AtXTH30	NP_174496
AtXTH31	NP_190085	AtXTH32	NP_181224
CsXTH1	CAD87535	FpXET1	CAC40807
FpXET2	CAC40808	FsXTH	CAA10231
HvXEA	P93671	HvXEB	P93672
NtEXT1	BAA32518	NtEXT2	BAA13163
NtXTH	ADV41673	PtXTH	XP_002318936
PtXTH1	ABK92523	PtXTH2	ABK95902
PtXTH3	ABK94576	PtXTH4	ABK92940
PtXTH5	ABK95370	PttXTH16-1	ABM91065
PttXTH16-3	ABM91070	PttXTH16-14	ABM91071
PttXTH16-17	ABM91073	PttXTH16-21	ABM91075
PttXTH16-26	ABM91063	PttXTH16-27	ABM91069
PttXTH16-30	ABM91074	PttXTH16-34	AAN87142
PttXTH16-35	ABL75361	PttXTH16-36	ABM91067
PttXTH16-39	ABM91072	RcXTH	XP_002526228
SlXTH1	BAA03923	SlXTH4	AAG43444
SlXTH5	AAS46240	SlXTH6	AAS46242
SlXTH8	BAA88668	SlXTH12	AAF17600
SpXTH1	BAE06063	TaXTH1	AAT94293
TaXTH3	AAT94295	TaXTH4	AAT94296
TaXTH5	AAT94297	VaXTH1	Q41638
VaXTH2	BAC03238	VvXTH	XP_002267890
ZmXTH1	NP_001105166	ZmXTH2	ACF85167
ZmXTH3	NP_001151500	ZmXTH4	NP_001130486
ZmXTH5	ADB54615	ZmXTH6	ACF88385

Protein sequences were obtained from NCBI RefSeq, GenBank database, and UniProtKB/Swiss-Prot database.

Supplementary Table S2. Primers used for quantitative real-time PCR.

Target	Primer name	Primer sequence (5’ to 3’)
PeXTH (in *P. euphratica*)	Forward primer	AAAGGGTCTGCGTGGGATG
	Reverse primer	CGGGAGGAGAAGTGGTTG
PeXTH (in tobacco)	Forward primer	TTAGCCAAGGCAAAGGCAAC
	Reverse primer	AGCCCAGTCATCAGCATTCC
PeACT7	Forward primer	ATGCTGCTAGGAGCCAGTGC
	Reverse primer	TTGTGCTCAGTGGTGGCTCTAC
NtEF1α	Forward primer	GCTGTGAGGGACATGCGTCAAA
	Reverse primer	GTAGTAGATATCGCGAGTACCACCA
NtXTH (D86730)	Forward primer	GCTAGTCACCACATCAAGTA
	Reverse primer	CTGAGTCTCCACCAACAAG
NtXTH (AB017025)	Forward primer	CAGAGCACGATGAGATAGAT
	Reverse primer	TGATGTGTAGGAGGCAGTA
PeXTH-1	Forward primer	GTGATTCTGCTGGAACTGT
	Reverse primer	GGTTCTCCTGTGGTGTTG
PeXTH-3	Forward primer	TCCACTACTATTCTGTCCTCT
	Reverse primer	CCAATCATCGGCATTCCA
PeXTH-4	Forward primer	CAGAGCACGATGAGATAGAT
	Reverse primer	TGATGTGTAGGAGGCAGTA

In this study the PCR conditions were: 95 ℃ for 5 min, followed by 34 cycles of 94 ℃ for 30 s, 55 ℃ for 30 s, and 72 ℃ for 30s, with a final step of 72 ℃ for 10 min.

Supplementary Table S3. The segregation ratio of kanamycin-resistant (Kan^R) to kanamycin-sensitive (Kan^S) seedlings among T_1 progeny of *PeXTH*-transgenic plants.

Line	Kan^R/Kan^S	Segregation ratio ($\chi^2_{0.05}$ test)
WT	0/107	0
L5	78/23	$3.4:1^{N.S.}$
L6	98/25	$3.9:1^{N.S.}$
L8	82/30	$2.7:1^{N.S.}$
L14	89/25	$3.6:1^{N.S.}$

N.S. indicates no significant difference between the transgene segregation ratio and the expected 3:1 Mendelian segregation for a single insertion site ($P > 0.05$).

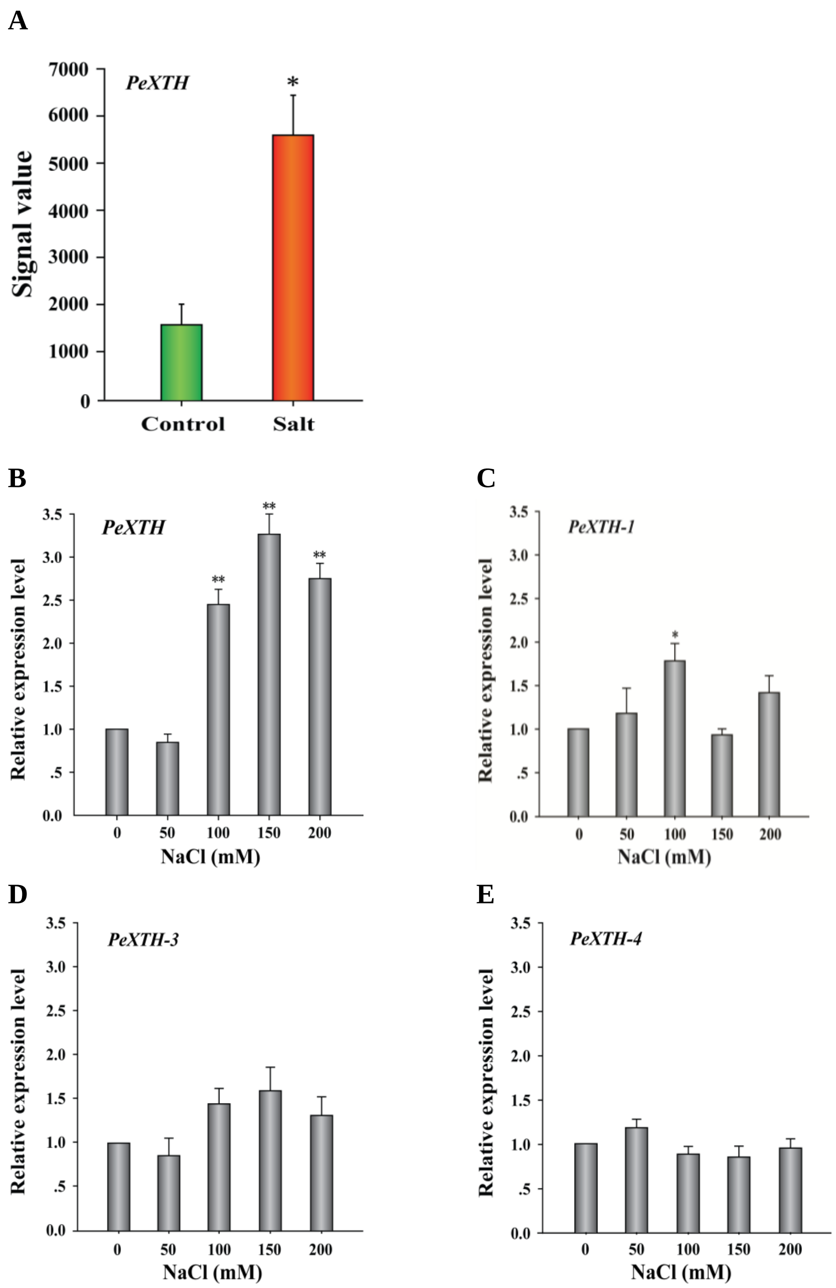

Supplementary Fig. S1. Expression profiles of *XTH* isoforms in *P. euphratica* leaves under salt stress. (A) Microarray analysis of *PeXTH* using the Affymetrix Poplar Array (Ding *et al.*, 2010; Affymetrix; Santa Clara, CA, USA). *PeXTH* gene (Probeset ID: PtpAffx.120153.1.S1-S-at; an *Arabidopsis thaliana* ortholog) was up-regulated in *P. euphratica* after exposure to 200 mM NaCl for 28 days. (B-E) Real-time quantitative PCR analysis of four *XTH* isoforms (*PeXTH*, *PeXTH-1*, *PeXTH-3*, and *PeXTH-4*). *P. euphratica* seedlings were subjected to 4 weeks of increasing NaCl, which started from 50 mM and increased stepwise by weekly 50 mM, reaching 200 mM NaCl in the fourth week. Total RNA was extracted from leaves of *P. euphratica*. Primers designed to target these *XTH* genes (Supplementary Table S2) were based on *P. trichocarpa* homologs obtained from the NCBI database (http://www.ncbi.nlm.nih.gov/). *ACT7* was used as an internal control. Each column is the mean of three individual plants and bars represent the standard error of the mean. * $P < 0.05$, ** $P < 0.01$ control vs. salt treatment.

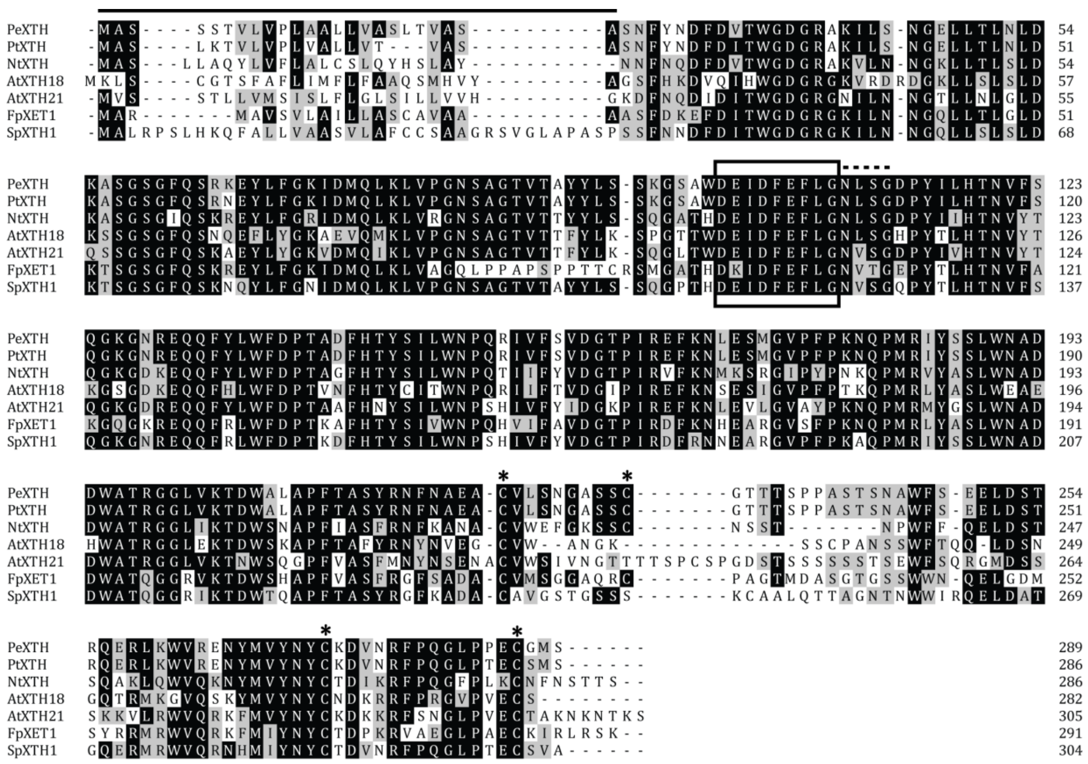

Supplementary Fig. S2. Multiple sequence alignment of PeXTH (xyloglucan endotransglucosylase/hydrolase from *P. euphratica*) with other XTHs from different species. The XTH sequences are from *Arabidopsis thaliana* (AtXTH18, AtXTH21), *Festuca pratensis (FpXET1), Nicotiana tabacum (NtXTH), Populus euphratica (PeXTH), Populus trichocarpa (PtXTH),* and *Sagittaria pygmaea (SpXTH1)*. The accession numbers of these sequences are provided in Supplementary Table S1. Black and grey shading indicate identical and conserved amino acid residues, respectively. Residues enclosed in a box indicate the conserved DEIDFEFLG catalytic domain of XTHs. The putative N-glycosylation motif immediately following the catalytic domain is marked by a dotted line. The four conserved Cys residues (C) are labeled by asterisk (*). The putative signal peptide sequence of PeXTH is marked by a solid line.

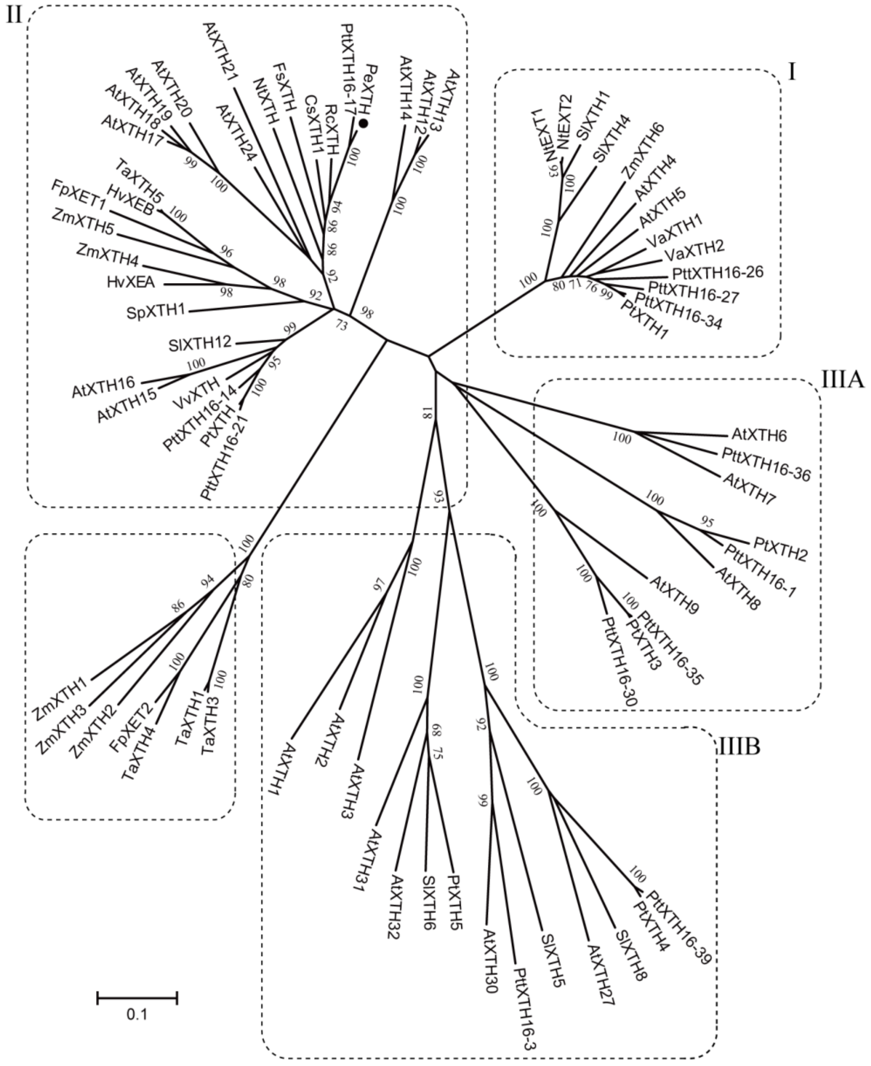

Supplementary Fig. S3. Phylogenetic relationships between PeXTH and other representative XTH proteins from different plant species. Seventy-three amino acid sequences of XTH (without signal peptides) were used for phylogenetic analysis. The numbers at the branches indicate bootstrap percentages (values < 70% are not shown). The different species are indicated as follows: At, *Arabidopsis thaliana*; Cs, *Cucumis sativus* (cucumber); Fp, *Festuca pratensis*; Fs, *Fagus sylvatica* (European beech); Hv, *Hordeum vulgare* (barley); Nt, *Nicotiana tabacum* (tobacco); Os, *Oryza sativa* (rice); Pe, *Populus euphratica*; Pt, *Populus trichocarpa*; Ptt, hybrid aspen *Populus tremula* L. × *P. tremuloides* Michx; Rc, *Ricinus communis* (castor); Sl, *Solanum lycopersicum* (tomato); Sp, *Sagittaria pygmaea*; Ta, *Triticum aestivum* (wheat); Va, *Vigna angularis* (azuki bean); Vv, *Vitis vinifera* (grape); Zm, *Zea mays* (maize). The accession numbers of the XTH sequences are provided in Supplementary Table S1.

A

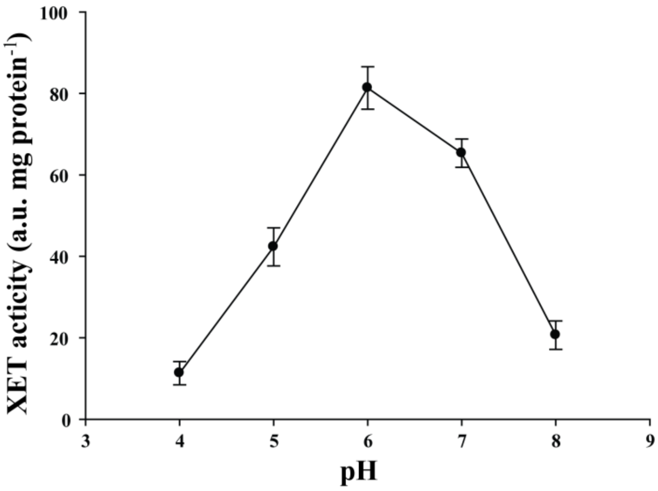

B

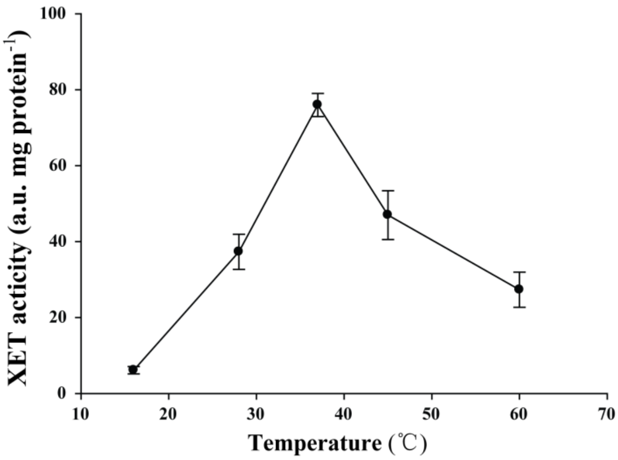

Supplementary Fig. S4. Effects of pH (A) and temperature (B) on xyloglucan endotransglucosylase (XET) activity of purified PeXTH protein. PeXTH protein was recombinantly produced using the prokaryotic expression system (*Escherichia coli* BL21) and purified with Ni-Sepharose media (GE Healthcare, USA). XET activity of purified PeXTH protein was tested at various pH values (4.0-8.0) and temperature (16-60°C). Each point is the mean of three independent replicates. Bars represent the standard error (SE) of the mean.

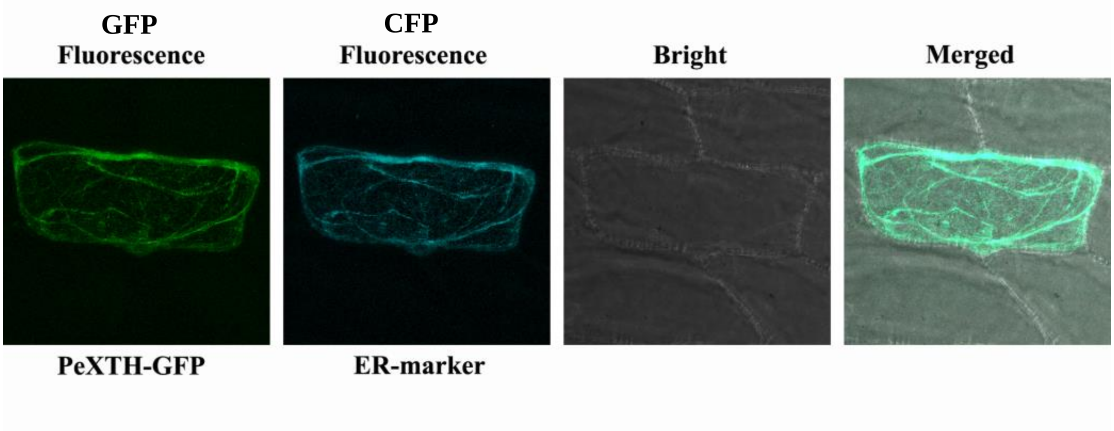

Supplementary Fig. S5. Co-localization of PeXTH–GFP with the endoplasmic reticulum marker (ER-ck *CD3-953*) in non-plasmolysed onion cells. Onion epidermal cells were bombarded with 5 μg of the recombinant plasmids (*PeXTH–GFP*) with the endoplasmic reticulum marker (cyan fluorescent protein (CFP), ER-ck *CD3-953*), using a biolistic PDS-1000/He particle delivery system (Bio-Rad, Hercules, CA, USA). GFP and CFP fluorescence was measured with Leica SP5 confocal microscope (Leica Microsystems GmbH, Wetzlar, Germany). The confocal settings were excitation at 488 nm (GFP) and 453 nm (CFP), and emission at wavelength of 510-535 nm (GFP) and 460-485 nm (CFP), respectively.

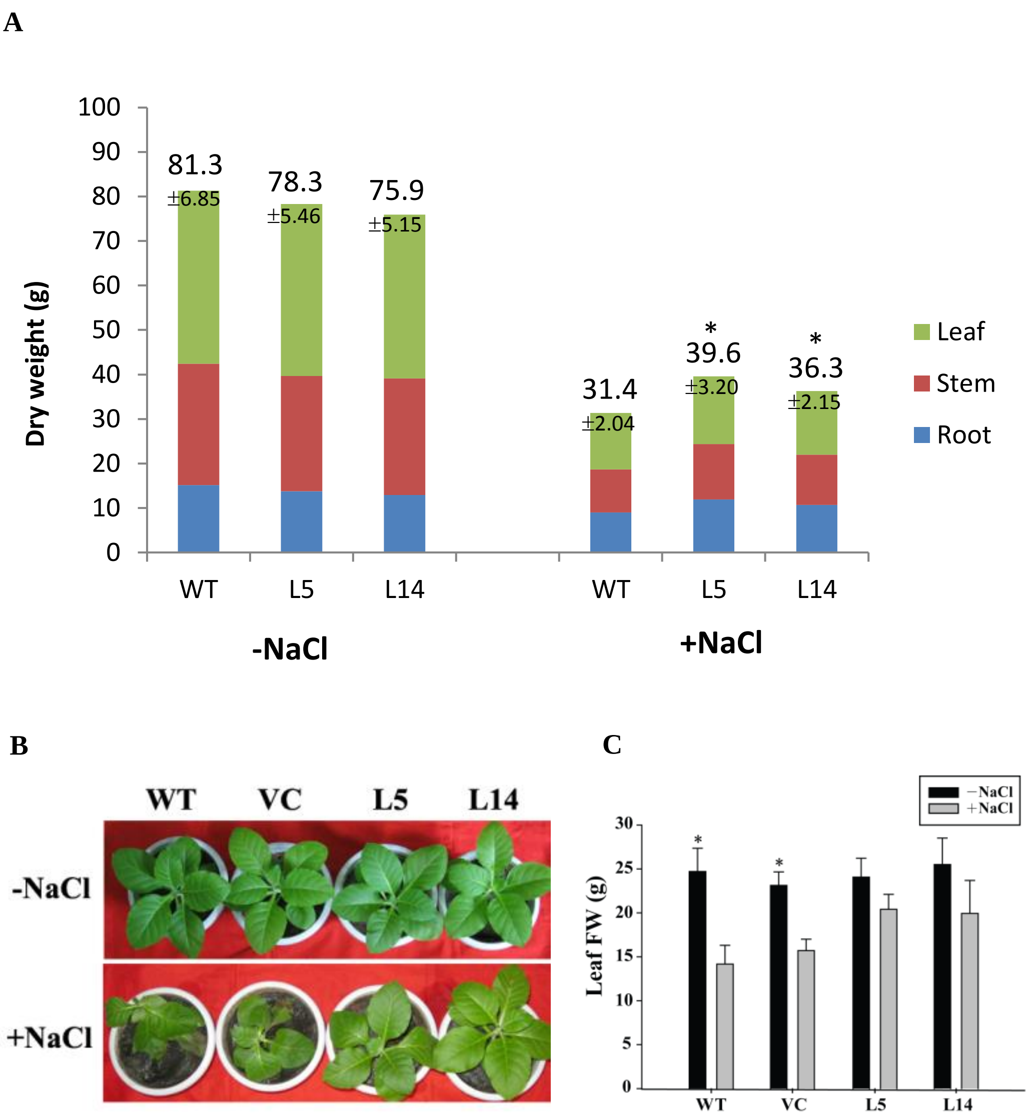

Supplemental Fig. S6. Salt tolerance of wild-type tobacco and *PeXTH*-transgenic plants grown in hydroponics and nursery soil supplemented or not with NaCl. (A) Long-term of hydroponic culture. Wild-type (WT) tobacco and *PeXTH*-overexpressing lines (L5 and L14) were subjected to 80 days of increasing salt stress. NaCl saline increased weekly from 50 to 200 mM, and then kept at 200 mM until the end of experiment. Dry weights of root, leaf, and stem are shown. Each column labeled with values (±SE) is the mean of three plants. * $P < 0.05$, WT vs. *PeXTH*-transgenic lines. (B-C) Soil culture. In this series, wild-type, vector control (VC), and the two transgenic lines (L5 and L14) germinated on half-strength MS medium, were grown in nursery soil for 30 days. Salt treatment was applied by top watering of 150 mM NaCl. Wilted leaves were only seen in WT and VC after 7 days of salt treatment (B). Leaves were harvested from control and salt-stressed plants and fresh weight (FW) were obtained (C). Each column is the mean of three tobacco plants. The bars indicate the standard error of the mean. * $P < 0.05$ control vs. salt treatment.

***NtXTH* gene (D86730)**

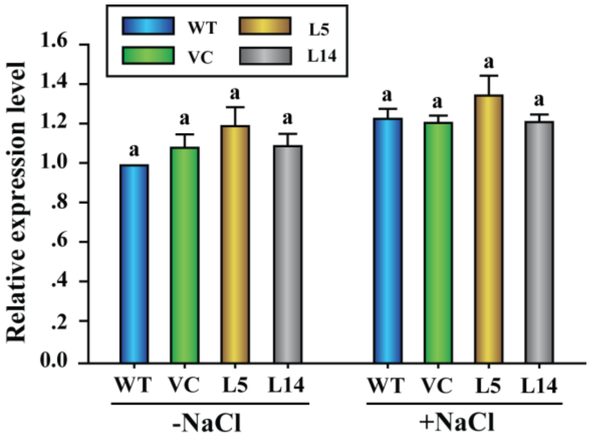

***NtXTH* gene (AB017025)**

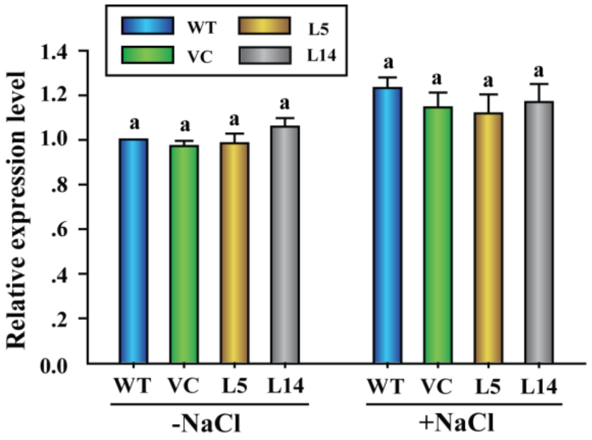

***PeXTH* gene**

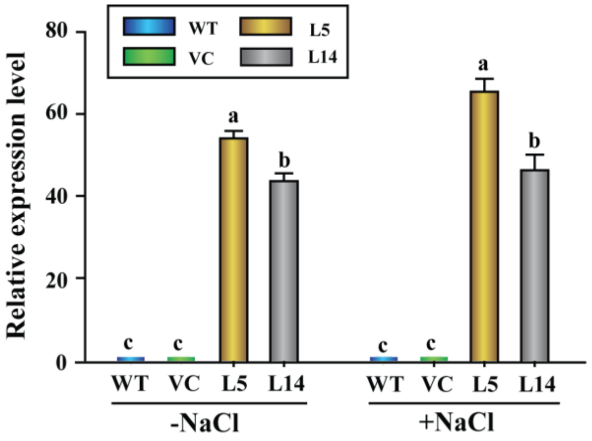

Supplementary Fig. S7. Expression of *PeXTH* gene and two tobacco-intrinsic *NtXTH* genes in wild-type (WT), vector control (VC) and *PeXTH*-transgenic tobacco plants (L5 and L14). Accession numbers of the two *NtXTH* genes are D86730 and AB017025 (Supplementary Table S2). Plants were acclimated to one-quarter-strength Hoagland nutrient solution for 7 days and then treated with 0 and 150 mM NaCl for additional 7 days. The expression levels were detected by real-time PCR analysis using the primers listed in Supplementary Table S2. *EF1α* was used as the internal control. The bars represent the standard error of the mean. Columns labeled with different letters indicate significant differences among the wild-type, vector control and transgenic lines at $P < 0.05$ under control (-NaCl) and salt treatment (+NaCl).
